# Postoperative radiotherapy with or without concurrent chemotherapy for oral squamous cell carcinoma in patients with three or more minor risk factors: a propensity score matching analysis

**DOI:** 10.1186/s13014-017-0910-0

**Published:** 2017-11-22

**Authors:** Kang-Hsing Fan, Yen-Chao Chen, Chien-Yu Lin, Chung-Jan Kang, Li-Yu Lee, Shiang-Fu Huang, Chun-Ta Liao, Shu-Hang Ng, Hung-Ming Wang, Joseph Tung-Chieh Chang

**Affiliations:** 1 Department of Radiation Oncology, Chang Gung Memorial Hospital at LinKou, Taoyuan, Taiwan; 2 Departments of Radiation Oncology, Chang Gung Memorial Hospital at Keelung, Keelung, Taiwan; 3 Department of Internal Medicine, Division of Hematology/Oncology, Chang Gung Memorial Hospital at LinKou, Taoyuan, Taiwan; 4 Departments of Otolaryncology-Head and Neck Surgery, Chang Gung Memorial Hospital at LinKou, Taoyuan, Taiwan; 5 Departments of Pathology, Chang Gung Memorial Hospital at LinKou, Taoyuan, Taiwan; 6 Departments of Diagnostic Radiology, Chang Gung Memorial Hospital at LinKou, Taoyuan, Taiwan; 7 Graduate Institute of Clinical Medical Science, School of Medicine, Chang Gung University, Taoyuan, Taiwan; 8 Department of Medicine, School of Medicine, Chang Gung University, Taoyuan, Taiwan; 9 Department of Radiation Oncology, Xiamen Chang Gung Memorial Hospital, Xiamen, Fujian China

**Keywords:** Head and neck cancer, Oral cavity cancer, Radiotherapy, Concurrent chemotherapy, Postoperative radiotherapy

## Abstract

**Background:**

To investigate the advantage of concurrent chemotherapy with postoperative radiotherapy (RT) of oral squamous cell carcinoma (OSCC) in patients with three or more minor risk factors.

**Methods:**

Minor risk factors included pT4 disease, pN1 disease, margin ≤ 4 mm, poor differentiation, perineural invasion, vessel or lymphatic invasion, and tumor invasion depth ≥ 11 mm. Surgery was the primary treatment, followed by RT or concurrent chemoradiation (CCRT). After propensity score matching, 34 patients in each treatment group were selected for comparison.

**Results:**

The median follow-up for living patients was 86.4 months (range: 47–189 months). The 5-year overall survival of the RT and CCRT groups was 35.3% and 67.2% (*p* = 0.018), respectively. The 5-year recurrence-free survival of the RT group and CCRT group was 42.6% and 75.4% (*p* < 0.01).

**Conclusion:**

Postoperative CCRT for patients with three or more minor risk factors increased recurrence-free and overall survival.

## Background

Treatment for advanced head and neck cancer typically includes a combination of different modalities. For patients who undergo surgery, adjuvant radiotherapy (RT) can reduce the risk of tumor recurrence when advanced features are noted [1–3]. The presence of nodal metastasis with extracapsular spreading and positive surgical margins are clear indications for postoperative radiotherapy (PORT) with concurrent chemotherapy [4, 5]. However, our previous study showed that the presence of three or more minor risk factors in pathological samples of oral squamous cell carcinoma (OSCC) was correlated with an inferior outcome after surgery and PORT. These minor risk factors include T4 disease, pathological N1 disease, a surgical margin ≤4 mm, poor differentiation, perineural invasion, vessel invasion, lymph invasion, and tumor invasion depth ≥ 11 mm. Tumor recurrence was increased compared with that in the control arms of the two randomized studies cited above [4–6]. Our previous study was based on the notion of risk accumulation, and other studies have also considered risk accumulation. Parsons et al. found that the number of indications for PORT was a predictor of locoregional recurrence for head and neck cancer [7]. Therefore, it is reasonable to hypothesize that the presence of multiple risk factors indicates the need for more intensive treatment. Thus, patients with three or more risk factors were directed to undergo postoperative concurrent chemoradiation (CCRT) after 2007. The present study was undertaken to analyze the treatment results.

## Methods

With the permission of the institutional review board, we retrieved clinical data on OSCC patients with three or more minor risk factors from the cancer registry from 1999 to 2009. After reviewing medical records and tumor board discussion records, 109 patients were selected. The exclusion criteria included the presence of (or no information regarding) positive resection margins, the presence of (or no information regarding) extracapsular spreading in metastatic nodes, a history of previous cancer, a second synchronous cancer, no standard neck dissection (at least supraomohyoid dissection), or any contraindication for CCRT recorded in the tumor board discussion. Tumor staging was based on the pathology findings and revised according to the 7th edition of the American Joint Committee on Cancer (AJCC) staging system [8].

All characteristics and treatment parameters were reviewed and recorded. Anemia was defined by hospital standard (hemoglobin < 13.5 g/dL in male and < 12 g/dL in female). Propensity-score matching was performed to reduce bias. R Statistical Software (version 3.2.4; R Foundation for Statistical Computing, Vienna, Austria) was used with Matchit package, and matching method was nearest-neighbor with 1 to 1 matching. Patients were divided into 2 group according to treatment method (postoperative RT or CCRT), 34 patients in each treatment group (RT and CCRT groups) were selected.

All patients received postoperative radiotherapy consisting of a conventional fractionated dose of 1.8 or 2 Gy at one fraction per day 5 days per week. A 6-MV photon beam was used for a total dose of 60 to 66 Gy. The initial treatment volume included the primary tumor bed with general margins and the regional cervical lymph nodes. Some patients received PORT by conventional field arrangement, which included a bilateral opposing field and a low anterior portal. The spinal cord was shielded after 46 to 46.8 Gy was delivered. Then, posterior and lower cervical lymph nodes were irradiated by an electron beam if necessary. Other patients received PORT by 3-dimensional conformal radiation therapy (3DCRT) or intensity modulation radiation therapy (IMRT). The dose delivered to the spinal cord and brain stem was limited to 50 Gy. Without violation of constraints for the brain stem and spinal cord, 95% of the clinical tumor volume and 90% of the planning treatment volume should be irradiated at 100% of the prescribed dose. After the administration of 46 to 50 Gy, the treatment area was reduced such that only the tumor bed and regions with metastatic nodes were irradiated.

Concurrent chemotherapy was cisplatin-based and was administered at either a low or high dose. For low-dose cisplatin, the prescribed dose was 40 to 50 mg/m^2^ administered every week or every other week, with or without an additional oral 5-fluorouracil prodrug [9, 10]. For high-dose cisplatin, the prescribed dose was 100 mg/m^2^ administered every 3 weeks. High-dose chemotherapy was typically administered to patients for two to three cycles, whereas low-dose chemotherapy was administered for four to six cycles.

The outcome measures included locoregional recurrence, distant metastasis, second primary cancer, and death. A re-staging study in patients with a recurrent tumor or a second primary cancer was used to define the tumor extension. Salvage treatment or best supportive care was given depending on the status of the disease and the patient. If a death occurred, the cause was reviewed in detail. Recurrence or a second primary cancer was verified by pathological examination or consequent clinical findings if no tissue was available. Second primary cancers and death unrelated to recurrence or complications were not considered treatment failure. The primary end points were death and tumor recurrence, and the secondary end points were locoregional recurrence and a second primary cancer. Survival was calculated from the date of radical surgery to the date of the event, which was defined as tumor recurrence or “death from disease” for recurrence-free survival (RFS) and death for overall survival (OS). Locoregional recurrence was defined as the event for locoregional recurrence-free survival (LRRFS). We used the Kaplan-Meier method for survival analysis and the log-rank test to determine whether there were significant differences between the patients with respect to the end points. The Cox regression model was used to perform the multivariate analysis. Correlations of each variable with the end points were evaluated by both univariate and multivariate analyses. Differences were considered significant when the *p*-value was < 0.05. The commercial statistics package PASW Statistics 18 (SPSS Inc., Chicago, IL) was used for the statistical analysis.

## Results

### Patient population

The patient ages ranged from 33 to 70 years, with a median of 53 years. A total of 60 (88.2%) patients were male, and eight (11.8%) patients were female. The most common subsite was the buccal mucosa (26, 38.3%), followed by the tongue (16, 23.5%), gums (15, 22.1%), retromolar trigone (8, 11.8%), mouth floor (2, 2.9%), and hard palate (one, 1.5%). A total of 3 (4.4%), 15 (22.1%), 7 (10.3%), and 43 (63.3%) patients had pathological stage T1, T2, T3, and T4 disease, respectively, and 27 (39.7%) patients had pathological N1 nodal metastasis. Table 1 lists the characteristics of all patients after propensity score matching. The differences between the CCRT and RT groups were not significant, with the exception of a greater number of patients with pathological N1 stage in the CCRT group (*p* = 0.046).

**Table 1:** Characteristics of all patients

Characteristic	Frequency in the CCRT group (%)	Frequency in the RT group (%)	*p*-value (2-sided)
Sex			
Male	28 (82.4%)	32 (94.1%)	0.259
Female	6 (17.6%)	2 (5.9%)	
ECOG performance			
0–1	33 (97.1%)	32 (94.1%)	1
2	1 (2.9%)	2 (5.9%)	
Age			
< 40 years	2 (5.9%)	1 (2.9%)	1
≧ 40 years	32 (94.1%)	33 (97.1%)	
Other comorbidities			
No	19 (55.9%)	26 (76.5%)	0.123
Yes	15 (44.1%)	8 (23.5%)	
Smoking			
Yes	27 (79.4%)	29 (85.3%)	0.752
No	7 (20.6%)	5 (24.6%)	
Alcohol			
Yes	25 (73.5%)	27 (79.4%)	0.776
No	9 (26.5%)	7 (20.6%)	
Betel quid			
Yes	21 (61.8%)	26 (76.5%)	0.294
No	13 (38.2%)	8 (23.5%)	
Anemia			
Yes	10 (29.4%)	10 (29.4%)	1.000
No	24 (70.6%)	24 (70.6%)	
Site^a^			
Tongue	9 (26.5%)	7 (20.6%)	0.738
Mouth floor	1 (2.9%)	1 (2.9%)	
Buccal mucosa	12 (35.3)	14 (41.2%)	
Gums	8 (23.5%)	7 (20.6%)	
Hard palate	1 (2.9%)	0	
Retromolar trigone	3 (8.8)	5 (14.7%)	
Differentiation			
Other	30 (88.2%)	28 (82.4%)	0.734
Poor	4 (11.8%)	6 (17.6%)	
Pathologic T stage			
T1–3	13 (38.2%)	12 (35.3%)	1
T4	21 (61.8%)	22 (64.7%)	
Pathologic N stage			
N0	16 (47.1%)	25 (73.5%)	0.046
N1	18 (52.9%)	9 (26.5%)	
Margin distance			
< 5 mm	19 (55.9%)	24 (70.6%)	0.314
≥ 5 mm	15 (44.1%)	10 (29.4%)	
Skin invasion			
Yes	6 (17.6%)	9 (26.5%)	0.56
No	28 (82.4%)	25 (73.5%)	
Bone invasion			
Yes	14 (41.2%)	16 (47.1%)	0.807
No	20 (58.8%)	18 (52.9%)	
Perineural invasion			
Yes	21 (61.8%)	22 (64.7%)	1
No	13 (38.2%)	12 (35.3%)	
Vascular invasion			
Yes	4 (11.8%)	1 (2.9%)	0.356
No	30 (88.2%)	33 (97.1%)	
Lymphatic invasion			
Yes	1 (2.9%)	1 (2.9%)	1
No	33 (97.1%)	33 (97.1%)	
Invasion depth of tumor			
< 10 mm	5 (24.6%)	2 (5.9%)	0.427
≥ 10	29 (75.4%)	32 (94.1%)	

^a^Sum was not 100% due to rounding

Abbreviations: *RT* radiotherapy, *CCRT* concurrent chemoradiation

### Radiotherapy and chemotherapy

A total of 42 (61.8%) patients began PORT within 6 weeks after radical surgery, and 61 (89.7%) patients completed PORT within 8 weeks. PORT was performed in 30 (44.1%) patients using the IMRT technique. One patient did not complete the entire radiotherapy course. This patient received CCRT and was the only patient who died from adverse events. A total of 23 (33.8%) patients received a total PORT dose of 66 Gy, and the other patients received a total dose of at least 60 but less than 66 Gy. In the CCRT group, 6 (8.8%) patients did not receive the desired cisplatin dose (200 mg/m^2^). Details of treatment-related variables of the RT and CCRT groups are listed in Table 2. Because the CCRT group was a modern cohort, IMRT was significantly more common in this group. Differences in other variables were reduced to insignificance after matching.

**Table 2:** Treatment variables of all patients

Characteristic	CCRT group (%)	RT group (%)	*p*-value
RT technique			
Other	13 (38.2%)	25 (73.5%)	< 0.01*
IMRT	21 (61.8%)	9 (26.5%)	
RT duration (days)			
≤ 8 weeks	32 (94.1%)	29 (85.3%)	0.427
> 8 weeks	2 (5.9%)	5 (24.7%)	
Time between OP & RT			
≤ 6 weeks	20 (58.8%)	22 (64.7%)	0.803
> 6 weeks	14 (41.2%)	12 (35.3%)	
RT dose			
< 6600 cGy	20 (58.8%)	25 (73.5%)	0.305
6600 cGy	14 (41.2%)	9 (26.5%)	

*Significant difference, *p* < 0.05

Abbreviations: *RT* radiotherapy, *IMRT* intensity-modulated radiotherapy, *OP* operation, *CCRT* concurrent chemoradiation

### Overall survival

**Fig. 1:**
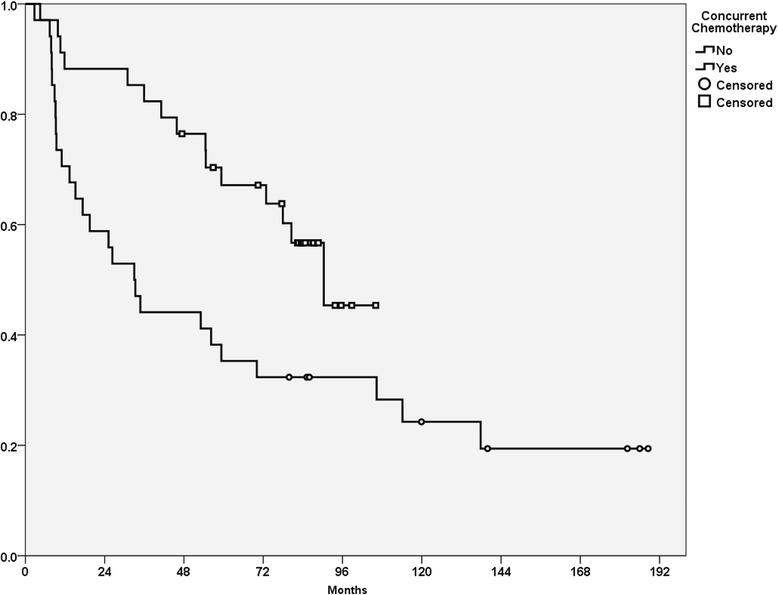
Overall survival curve for patients receiving or not receiving concurrent chemotherapy in the CRT and RT cohorts (*p* < 0.01)

At the time of the analysis, 41 patients had died. The cause of death was cancer recurrence in 26 cases, treatment-related adverse events in one case (pneumonia), a second primary cancer in 11 cases, and non-cancer disease in 3 cases. The median follow-up time among survivors were 130 months and 86 months for RT group and CCRT group, respectively. The 5-year OS rate was 51.2%, and the median survival was 59 months. The median survival of the CCRT and RT group were 79.5 months and 33 months, respectively. The 5-year OS rates of the CCRT and RT group were 67.2 and 35.3%, respectively (*p* = 0.02, Fig. 1). In the univariate analysis, the absence of chemotherapy and presence of anemia were correlated with poor OS (*p* < 0.05, Table 3). In the multivariate analysis, betel quid chewing, anemia, tumor invasion depth ≥ 11 mm, and concurrent chemotherapy were independent prognostic factors for OS (*p* < 0.05, Table 4).

**Table 3:** Overall and recurrence-free survival according to patient characteristics and treatment variables

Characteristic	5-year overall survival (*p*-value)	5-year recurrence-free survival
Sex		
Male	51.4% (0.539)	56.7% (0.826)
Female	50%	75%
Age		
< 40 years	100% (0.136)	100% (0.19)
≧ 40 years	49.1%	56.9%
Smoking		
Yes	51.5% (0.536)	57.2% (0.91)
No	50%	66.7%
Alcohol		
Yes	55.5% (0.311)	57.6% (0.575)
No	35.7%	72.7%
Betel quid		
Yes	42.2% (0.068)	50.8% (0.154)
No	71.1%	75.9%
Anemia		
Yes	29.2% (0.002)*	54% (0.106)
No	60.2%	61%
Differentiation		
Poor	40% (0.752)	30% (0.03)*
Well or moderate	53.1%	64%
pT stage		
pT4	52.9% (0.978)	64% (0.461)
pT1–3	48%	50%
pN stage		
N1	55.3% (0.481)	62.7% (0.34)
N0	48.4%	53.8%
Surgical margin		
≤ 4 mm	48.2% (0.791)	59.8% (0.587)
> 4 mm	56%	57.6%
Skin invasion		
Yes	59.3% (0.485)	64.6% (0.428)
No	48.9%	57.1%
Bone invasion		
Yes	46.4% (0.44)	55.4% (0.396)
No	55%	61.7%
Perineural invasion		
Yes	51.2% (0.953)	58.8% (0.723)
No	50.2%	59.2%
Vascular invasion		
Yes	60% (0.444)	60% (0.785)
No	48.8%	57%
Lymphatic invasion		
Yes	50% (0.948)	50% (0.752)
No	51.2%	59.1%
Invasion depth		
≥ 11 mm	52.1% (0.432)	69.2% (0.177)
< 11 mm	42.9%	57.1%
RT technique		
Other	47.2% (0.623)	51.7% (0.489)
IMRT	56.1%	78.3%
RT duration		
> 8 weeks	28.6% (0.07)	28.6% (0.063)
≤ 8 weeks	53.7%	62.3%
Time between OP & RT		
> 6 weeks	42% (0.192)	48.2% (0.38)
≤ 6 weeks	57%	65.5%
RT Dose		
< 6600 cGy	39.2% (0.065)	50.8% (0.191)
6600 cGy	73.9%	73.7%
Concurrent chemotherapy		
No	35.3% (0.018)*	426% (0.009)*
Yes	67.2%	75.4%

*Statistically significant in the multivariate analysis, *p* < 0.05

Abbreviations: *RT* radiotherapy, *IMRT* intensity-modulated radiotherapy, *OP* operation

**Table 4:** Multivariate analysis of overall and recurrence-free survival according to patient characteristics and treatment variables

Characteristic	Overall survival *p*-value	Overall survival HR (95% CI)	Recurrence-free survival *p*-value	Recurrence-free survival HR (95% CI)
Chemotherapy	0.017	0.426 (0.212–0.858)	0.002	0.248 (0.103–0.596)
Betel quid	0.004	3.951 (1.567–9.966)	NS	NS
Anemia	0.003	3.1 (1.488–6.461)	NS	NS
Pathological T4	NS	NS	0.016	0.355 (0.153–0.826)
Invasion depth ≥ 11 mm	0.007	0.187 (0.055–0.632)	< 0.001	0.077 (0.02–0.303)
Interval between surgery and PORT	NS	NS	0.003	3.872 (1.582–9.474)

Abbreviation: *HR* hazard ratio, *CI* confidence interval, *PORT* postoperative radiotherapy, *NS* not significant

### Recurrence-free survival

**Fig. 2:**
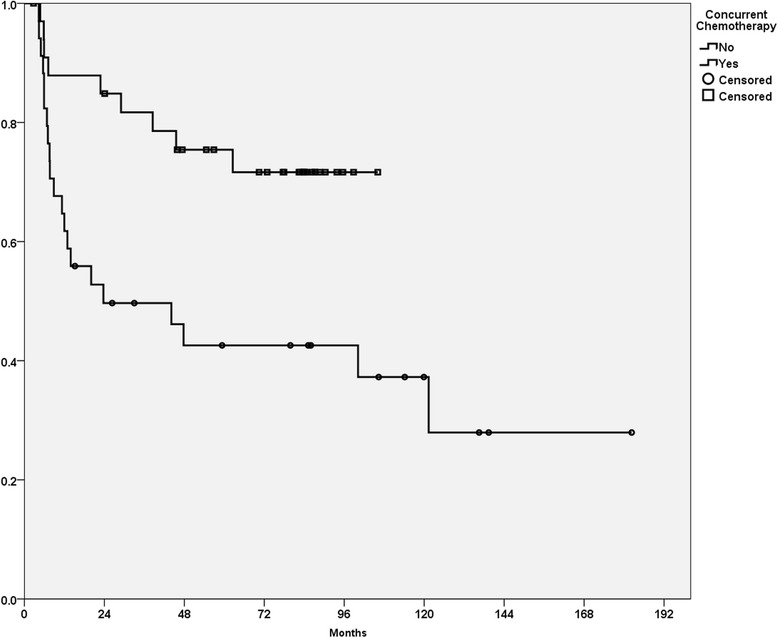
Recurrence-free survival curve for patients irradiated with or without concurrent chemotherapy in the CRT and RT cohorts (*p* < 0.01)

A total of 30 patients had documented disease recurrence. The 5-year RFS rate of all patients was 57.7%. Local recurrence (16) was the most common first recurrence pattern, followed by nodal (6), distant (4), both local and regional (1), and local/regional/distant all together (1). Four patients were salvaged by surgery and/or radiotherapy. The CCRT group had a significantly higher 5-year RFS rate than the RT group (75.4% vs. 42.6%, *p* < 0.01, Fig. 2). The absence of chemotherapy and poorly differentiated histology were significantly correlated with poor RFS (*p* < 0.05) in the univariate analysis (Table 3). In the multivariate analysis, tumor invasion depth ≥ 11 mm, pathological T4 disease, interval between surgery and PORT of more than 6 weeks, concurrent chemotherapy, and a habit of betel quid chewing were independent poor prognostic factors (*p* < 0.05, Table 4).

### Lethal adverse events, second primary cancers, and other death events

One patient died from acute adverse events. The mortality rate was 2.8% among patients who received CCRT. Fifteen patients developed second primary cancers during the follow-up period. Head and neck cancers were the most common type and occurred in 9 patients. Additionally, 2 patients developed lung cancer, 2 developed esophageal cancer, one developed prostate cancer, and one developed acute myelocytic leukemia during the follow-up period. Eight and 7 patients in the CCRT and RT groups, respectively, developed a second primary cancer, and the risk of developing a second primary cancer was similar between the two groups (chi-square test, two-tailed, *p* = 0.9).

## Discussion

Radiotherapy is an important treatment for head and neck cancers. Indications for PORT are based on specific findings obtained from pathology samples. Clinical trials have confirmed the role of postoperative CCRT in the treatment of head and neck cancers with positive resection margins or lymph node metastases with extracapsular spreading [4, 5]. However, according to previous studies, a higher tumor recurrence rate is also associated with the presence of three or more risk factors other than a positive resection margin or extracapsular spreading [6]. Since this finding, a shift in the treatment protocol from RT to CCRT was proposed. Fortunately, as demonstrated in the current study, the treatment result was significantly improved by CCRT.

Various methods are used to classify the risk recurrence and assign the appropriate treatment for head and neck cancer. Dominant prognostic factors, such as a positive resection margin and extracapsular spread, can indicate the need for a different treatment. Additionally, developing a prediction model or a nomogram using multiple prognostic factors can serve as another method [11]. Risk factor clustering is another means by which tumor recurrence risks can be classified. The presence of a greater number of risk factors correlates with an increased risk of tumor recurrence in retrospective analyses [7, 12]. A randomized trial of dose escalation for head and neck cancer also found that the risk of tumor recurrence increased with the clustering of two or more prognostic factors [13]. Our previous study showed the same result in OSCC while excluding disease with positive resection margin or extracapsular spread. Thus, clustering of 3 or more minor risk factors is an indication of a higher tumor recurrence risk. The present study took a step further to review the results of a new treatment protocol.

The use of CCRT to improve the treatment outcome of OSCC patients presenting with three or more minor risk factors is reasonable. CCRT reduces the risk of death from head and neck cancer, [14] and randomized trials have demonstrated that concurrent chemotherapy reduces locoregional recurrence rather than distant metastasis [4, 5] A previous study examining the effect of three or more minor risk factors reported that only 4% of all cases of first tumor recurrence involved distant metastasis [6]. Due to the high probability of locoregional recurrence, CCRT is likely to be beneficial. The current study revealed that CCRT greatly improves the outcomes of OSCC patients with three or more minor risk factors.

In the OS analysis, betel quid chewing was correlated with a lower OS. It was not the traditional prognostic factor which is related to treatment variables or disease status. Betel quid is a strong OSCC carcinogen [15]. It is reasonable that betel quid chewing was correlated with more death events when 11 of 41 deaths were caused by a second primary cancer, and OSCC was the most common second primary cancer in this cohort.

Since treatment principle was changed with time and this study included patients from a long period of time, treatment techniques were different between groups and some clinical information was not available. In CCRT group, IMRT were more commonly used, and the median follow-up period among survivors was shorter. The data of human papillomavirus (HPV) prevalence was lacking in most of patients. Currently, IMRT has been approved that it significantly reduces the risk of toxicities for patients with head and neck cancer. But the benefit of better tumor control was only shown in radiotherapy for nasopharyngeal cancer [16]. Two randomized trials targeting head and neck cancer did not show any benefit from IMRT in tumor control and overall survival [17, 18]. Therefore, we believe that most of improvement in cancer control still came from concurrent chemotherapy. Although HPV status were unknown in most of the patients, but the role of HPV infection in oral cavity cancer is still controversial. Chung et al. showed that HPV status correlated with prognosis only in oropharyngeal cancer [19]. Other studies focusing on OSCC and HPV status used different detection method and both better and worse prognosis correlated HPV status were reported [20, 21]. Therefore, the result of current study should not be affected significantly without data of HPV infection.

When used with concurrent chemotherapy, other treatment factors did not alter the treatment result. However, CCRT was more toxic to patients. In the current study, CCRT resulted in a mortality rate of 2.8%, which was comparable to the rate in postoperative CCRT arms in randomized trials [4, 5]. However, less toxic but equally effective regimens should be investigated. In a randomized trial of head and neck cancer, adding cetuximab to radiotherapy reduced locoregional recurrence and mortality without increasing toxicity [22]. Unfortunately, no published results have shown that cetuximab has efficacy equal to or better than that of cisplatin. One retrospective study even reported that cetuximab is inferior to cisplatin with respect to tumor control and survival [23]. Another phase II randomized trial showed that, compared to cisplatin, cetuximab concomitant to RT lowered compliance and increased acute toxicity rates [24]. Although there is no study directly comparing cetuximab and cisplatin concomitant to postoperative RT, one randomized trial has approved that adding Cetuximab to cisplatin-based CCRT did not improve the treatment result [25]. That may also imply that Cetuximab is not effective in combination with CCRT. Before the efficacy of Cetuximab was approved by randomized trials, replacing cisplatin with cetuximab should be avoided.

## Conclusion

For patients with 3 or more minor risk factors, postoperative CCRT reduced the risk of tumor recurrence and increased OS compared with those in both the RT cohort and patients who refused chemotherapy in the CCRT group. The presence of three or more minor risk factors should be considered an indication for postoperative CCRT. A prospective, randomized trial may provide more unbiased evidence.

## Abbreviation

3DCRT: 3-dimensional conformal radiation therapy
AJCC: American Joint Committee on Cancer
CCRT: Concurrent chemoradiation
Gy: Gray
HPV: Human papillomavirus
IMRT: Intensity modulation radiation therapy
MV: Megavoltage
OS: Overall survival
OSCC: Oral squamous cell carcinoma
PORT: Postoperative radiotherapy
RFS: Recurrence-free survival
RT: Radiotherapy

## References

[1] Fletcher GH, Evers WT (1970). Radiotherapeutic management of surgical recurrences and postoperative residuals in tumors of the head and neck. Radiology.

[2] Kramer S, Gelber RD, Snow JB, Marcial VA, Lowry LD, Davis LW, Chandler R (1987). Combined radiation therapy and surgery in the management of advanced head and neck cancer: final report of study 73-03 of the radiation therapy oncology group. Head Neck Surg.

[3] Dimery IW, Hong WK (1993). Overview of combined modality therapies for head and neck cancer. J Natl Cancer Inst.

[4] Bernier J, Domenge C, Ozsahin M, Matuszewska K, Lefebvre JL, Greiner RH, Giralt J, Maingon P, Rolland F, Bolla M (2004). Postoperative irradiation with or without concomitant chemotherapy for locally advanced head and neck cancer. N Engl J Med.

[5] Cooper JS, Pajak TF, Forastiere AA, Jacobs J, Campbell BH, Saxman SB, Kish JA, Kim HE, Cmelak AJ, Rotman M (2004). Postoperative concurrent radiotherapy and chemotherapy for high-risk squamous-cell carcinoma of the head and neck. N Engl J Med.

[6] Fan KH, Wang HM, Kang CJ, Lee LY, Huang SF, Lin CY, Chen EY, Chen IH, Liao CT, Chang JT (2010). Treatment results of postoperative radiotherapy on squamous cell carcinoma of the oral cavity: coexistence of multiple minor risk factors results in higher recurrence rates. Int J Radiat Oncol Biol Phys.

[7] Parsons JT, Mendenhall WM, Stringer SP, Cassisi NJ, Million RR (1997). An analysis of factors influencing the outcome of postoperative irradiation for squamous cell carcinoma of the oral cavity. Int J Radiat Oncol Biol Phys.

[8] Edge SBBD, Compton CC, Fritz AG, Greene FL, Trotti A (2010). AJCC cancer staging manual.

[9] Wang HM, Wang CS, Chen JS, Chen IH, Liao CT, Chang TC (2002). Cisplatin, tegafur, and leucovorin: a moderately effective and minimally toxic outpatient neoadjuvant chemotherapy for locally advanced squamous cell carcinoma of the head and neck. Cancer.

[10] Bachaud JM, Cohen-Jonathan E, Alzieu C, David JM, Serrano E, Daly-Schveitzer N (1996). Combined postoperative radiotherapy and weekly cisplatin infusion for locally advanced head and neck carcinoma: final report of a randomized trial. Int J Radiat Oncol Biol Phys.

[11] Gross ND, Patel SG, Carvalho AL, Chu PY, Kowalski LP, Boyle JO, Shah JP, Kattan MW (2008). Nomogram for deciding adjuvant treatment after surgery for oral cavity squamous cell carcinoma. Head Neck.

[12] Hinerman RW, Mendenhall WM, Morris CG, Amdur RJ, Werning JW, Villaret DB (2004). Postoperative irradiation for squamous cell carcinoma of the oral cavity: 35-year experience. Head Neck.

[13] Peters LJ, Goepfert H, Ang KK, Byers RM, Maor MH, Guillamondegui O, Morrison WH, Weber RS, Garden AS, Frankenthaler RA (1993). Evaluation of the dose for postoperative radiation therapy of head and neck cancer: first report of a prospective randomized trial. Int J Radiat Oncol Biol Phys.

[14] Pignon JP, le Maitre A, Maillard E, Bourhis J, Group M-NC: Meta-analysis of chemotherapy in head and neck cancer (MACH-NC): an update on 93 randomised trials and 17,346 patients. Radiotherapy Oncol 2009, 92(1):4-14. 10.1016/j.radonc.2009.04.014 19446902

[15] Ko YC, Huang YL, Lee CH, Chen MJ, Lin LM, Tsai CC (1995). Betel quid chewing, cigarette smoking and alcohol consumption related to oral cancer in Taiwan. J. Oral Pathol. Med..

[16] Peng G, Wang T, Yang KY, Zhang S, Zhang T, Li Q, Han J, Wu G (2012). A prospective, randomized study comparing outcomes and toxicities of intensity-modulated radiotherapy vs. conventional two-dimensional radiotherapy for the treatment of nasopharyngeal carcinoma. Radiother Oncol.

[17] Nutting CM, Morden JP, Harrington KJ, Urbano TG, Bhide SA, Clark C, Miles EA, Miah AB, Newbold K, Tanay M (2011). Parotid-sparing intensity modulated versus conventional radiotherapy in head and neck cancer (PARSPORT): a phase 3 multicentre randomised controlled trial. Lancet Oncol.

[18] Gupta T, Agarwal J, Jain S, Phurailatpam R, Kannan S, Ghosh-Laskar S, Murthy V, Budrukkar A, Dinshaw K, Prabhash K (2012). Three-dimensional conformal radiotherapy (3D-CRT) versus intensity modulated radiation therapy (IMRT) in squamous cell carcinoma of the head and neck: a randomized controlled trial. Radiother Oncol.

[19] Chung CH, Zhang Q, Kong CS, Harris J, Fertig EJ, Harari PM, Wang D, Redmond KP, Shenouda G, Trotti A (2014). p16 protein expression and human papillomavirus status as prognostic biomarkers of nonoropharyngeal head and neck squamous cell carcinoma. J Clin Oncol.

[20] Chen YW, Kao SY, Yang MH (2012). Analysis of p16 (INK4A) expression of oral squamous cell carcinomas in Taiwan: prognostic correlation without relevance to betel quid consumption. J Surg Oncol.

[21] Lee LA, Huang CG, Liao CT, Lee LY, Hsueh C, Chen TC, Lin CY, Fan KH, Wang HM, Huang SF (2012). Human papillomavirus-16 infection in advanced oral cavity cancer patients is related to an increased risk of distant metastases and poor survival. PLoS One.

[22] Bonner JA, Harari PM, Giralt J, Azarnia N, Shin DM, Cohen RB, Jones CU, Sur R, Raben D, Jassem J (2006). Radiotherapy plus cetuximab for squamous-cell carcinoma of the head and neck. N Engl J Med.

[23] Koutcher L, Sherman E, Fury M, Wolden S, Zhang Z, Mo Q, Stewart L, Schupak K, Gelblum D, Wong R (2011). Concurrent cisplatin and radiation versus cetuximab and radiation for locally advanced head-and-neck cancer. Int J Radiat Oncol Biol Phys.

[24] Magrini SM, Buglione M, Corvo R, Pirtoli L, Paiar F, Ponticelli P, Petrucci A, Bacigalupo A, Crociani M, Lastrucci L (2016). Cetuximab and radiotherapy versus Cisplatin and radiotherapy for locally advanced head and neck cancer: a randomized phase II trial. J Clin Oncol.

[25] Ang KK, Zhang Q, Rosenthal DI, Nguyen-Tan PF, Sherman EJ, Weber RS, Galvin JM, Bonner JA, Harris J, El-Naggar AK (2014). Randomized phase III trial of concurrent accelerated radiation plus cisplatin with or without cetuximab for stage III to IV head and neck carcinoma: RTOG 0522. J Clin Oncol.

